# Sublingual immunization with recombinant adenovirus encoding SARS-CoV spike protein induces systemic and mucosal immunity without redirection of the virus to the brain

**DOI:** 10.1186/1743-422X-9-215

**Published:** 2012-09-21

**Authors:** Byoung-Shik Shim, Konrad Stadler, Huan Huu Nguyen, Cheol-Heui Yun, Dong Wook Kim, Jun Chang, Cecil Czerkinsky, Man Ki Song

**Affiliations:** 1 Laboratory Sciences Division, International Vaccine Institute, Seoul, 151-919, Republic of Korea; 2 Department of Agricultural Biotechnology and Research Institute for Agriculture and Life Sciences, and the Center for Agricultural Biomaterials, and Center for Food Safety and Toxicology, Seoul National University, Seoul, 151-921, Republic of Korea; 3 Department of Pharmacy, College of Pharmacy, Hanyang University, Kyeonggi-do, 426-791, Republic of Korea; 4 College of Pharmacy, Ewha Womans University, 11-1 Dae-Hyun Dong, Seo-Dae-Mun Gu, Seoul, 120-750, Republic of Korea

**Keywords:** Recombinant adenovirus, Sublingual administration, Severe acute respiratory syndrome, Mucosa, T cell, IgA

## Abstract

**Background:**

Sublingual (s.l.) administration of soluble protein antigens, inactivated viruses, or virus-like particles has been shown to induce broad immune responses in mucosal and extra-mucosal tissues. Recombinant replication-defective adenovirus vectors (rADVs) infect mucosa surface and therefore can serve as a mucosal antigen delivery vehicle. In this study we examined whether s.l. immunization with rADV encoding spike protein (S) (rADV-S) of severe acute respiratory syndrome-associated coronavirus (SARS-CoV) induces protective immunity against SARS-CoV and could serve as a safe mucosal route for delivery of rADV.

**Results:**

Here, we show that s.l. administration of rADV-S induced serum SARS-CoV neutralizing and airway IgA antibodies in mice. These antibody responses are comparable to those induced by intranasal (i.n.) administration. In addition, s.l. immunization induced antigen-specific CD8^+^ T cell responses in the lungs that are superior to those induced by intramuscular immunization. Importantly, unlike i.n. administration, s.l. immunization with rADV did not redirect the rADV vector to the olfactory bulb.

**Conclusion:**

Our study indicates that s.l. immunization with rADV-S is safe and effective in induction of a broad spectrum of immune responses and presumably protection against infection with SARS-CoV.

## Background

The majority of microbial pathogens enter their hosts through a mucosal site; hence, effective vaccines should elicit immune responses at the site of infection [1,2]. Ideally, vaccines against pathogens such as severe acute respiratory syndrome-associated coronavirus (SARS-CoV) which infects the airways should elicit immune responses in the mucosa of the respiratory tract [3]. Although mucosal application of vaccines is attractive for many reasons, only few mucosal vaccines [most of them are given by the oral route and only one intranasal (i.n.) live-attenuated influenza vaccine] have been approved for use in humans. Because oral administration of vaccines has been proven difficult for inducing immune responses in the respiratory tract [2], i.n. delivery of vaccines has been selected as an attractive alternative to injection. While i.n. vaccination elicits strong local and systemic immune responses, concerns about its safety have been raised following reports of unacceptable neurological side-effects associated with retrograde transport of antigens or adjuvants through the olfactory epithelium [4-7].

Replication-defective adenovirus (rADV) vectors are among the most attractive vectors for delivery of foreign antigens [8-20]. ADVs infect their host through the airway epithelium, and replicate in mucosal tissues of the respiratory tracts [21]. In a number of study models, mucosal vaccination with ADV has been shown to be effective at producing antibody (Ab) and T cell responses at the site of immunization [20,22,23]. However, when applied intranasally, ADV can enter the central nervous system (CNS) through binding to olfactory neurons [7,24,25]. The concerns call for alternative delivery routes.

The sublingual (s.l.) route has been extensively used for administration of immunotherapeutic allergens as a modality to induce suppression of type I allergic responses [26-29]. We have shown that s.l. administration of a prototype soluble protein antigen with cholera toxin (CT) adjuvant could induce Ab and cytotoxic T-lymphocyte (CTL) responses comparable to those seen after i.n. immunization [30]. In addition, s.l. administration of live influenza virus protected mice against influenza virus challenge without redirecting the immunizing virus to the CNS [31]. More recently, it has been shown that s.l. administration of rAd5 vectors encoding HIV proteins induced significant antigen-specific humoral [32] and cellular immune responses [33], indicating that s.l. route is suitable for rADV vaccines.

SARS-CoV is an enveloped virus containing a large single-strand RNA genome with positive orientation. The club-shaped peplomers radiating outwards from the viral envelope are composed of oligomeric forms of the ~180 kDa viral spike (S) glycoprotein. The S protein not only contains the receptor binding site and the putative fusion peptide, but it is also a major antigenic determinant of the virus and Abs targeting this protein neutralize the virus *in vitro* and *in vivo*[34-36].

In this study, we explored the suitability of the s.l. route for administering a replication-defective ADV encoding truncated S protein (rADV-S) lacking cytoplasmatic and transmembrane domains. The immune responses induced upon s.l. immunization with rADV-S were compared to those induced by i.n. and intramuscular (i.m.) routes. We found that s.l. delivery of rADV-S induced systemic and mucosal Abs, CD8^+^ T cell responses. Importantly, our immunization strategy generated SARS-CoV neutralizing antibodies (nAbs) at the titers that are presumably protective against the infection. In addition, we confirmed that s.l., in contrast to i.n. administration, did not redirect rADV to the olfactory bulb.

## Results

### Characterization of rADV expressing SARS-CoV S protein

**Figure 1:**
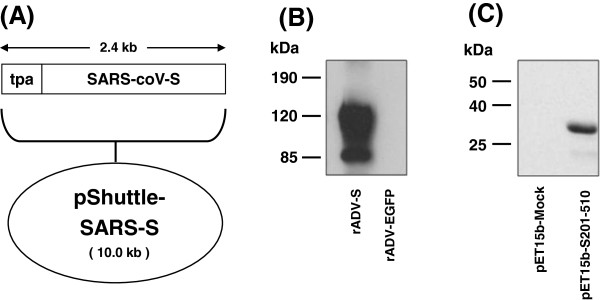
**Construction of rADV vector expressing SARS-CoV S glycoprotein and expression of S proteins in 293 T cells and bacteria.** (**A**) The gene encoding codon-optimized SARS-S protein without helical regions, transmembrane domain and cytoplasmic domain was inserted into pShuttle vector to construct the rADV expressing the SARS-S protein. (**B**) 293 T cells were infected with rADV-S or rADV-EGFP and the S protein in the cell lysate was detected by Western blot. (**C**) DNA for SARS-S protein (amino acids 201–510) was inserted into pET15b vector to express recombinant S protein from *E. coli*. The protein was purified by His-tag affinity chromatography and detected by Western blot.

To confirm the expression of S protein *in vitro*, 293 cells were infected with rADV-S at 20 multiplicity of infection (MOI) for 48 hrs. Cell lysates and culture supernatants were collected and analyzed by Western blot. As shown in Figure 1B, a specific ~ 120 kDa protein band corresponding to the predicted size of the S protein, was observed in rADV-S infected 293 cells but not in rADV-EGFP infected cells. The portion of the S1 domain (S201-510) expressed in and purified from *E. coli* was used as coating antigen in ELISA. The purified protein was confirmed by Western blot using rabbit anti-SARS-S1 Ab (Figure 1C).

### S.l. Administration of rADV induced mucosal Ab responses

**Figure 2:**
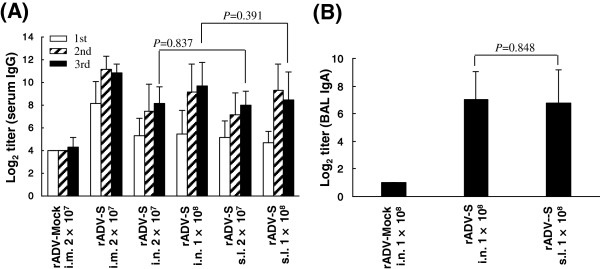
**SARS-CoV S-specific humoral immune responses in the immunized mice.** Mice were immunized with rADV-S by s.l., i.n., or i.m. route. Sera were collected two weeks after each vaccination and BAL was collected two weeks after the last immunization. S-specific IgG titers in sera (**A**) and S-specific IgA titers in BAL (**B**) were determined by ELISA. The results are expressed as the means + SD. The data are representative of three separate experiments.

To compare the immune responses induced by different delivery routes, we immunized s.l., i.n., or i.m. each group of BALB/c mice three times 14 days apart with either 2 × 10^7^ or 1 × 10^8^ plaque-forming unit (PFU) of rADV-S. Sera from the animals were collected 2 weeks after each immunization and tested for S protein-specific IgG by ELISA. IgG titers were detected as soon as 2 weeks after the first immunization regardless of delivery route. However, i.m. immunization induced the highest S protein-specific IgG titers in the sera as compared to i.n. and s.l. immunizations (Figure 2A). The peak of IgG titers was reached in all groups upon second immunization as third immunization did not significantly increase the IgG titers.

Since SARS-CoV infects mucosa of the lungs, an effective vaccination strategy should induce specific immune response in the lungs, the site of infection. I.n. administration is well recognized for excellent induction of immune responses in mucosal compartments of the respiratory tract [37,38]. We asked whether s.l. immunization with rADV-S induces antigen-specific Ab in the lungs. We examined the level of IgA specific for SARS-S protein in Bronchoalveolar lavages (BAL) of mice upon s.l. immunization. As shown in Figure 2B, significant level of IgA specific for SARS-S protein was observed in BAL of s.l. immunized mice 2 weeks after the third immunization. The IgA level is comparable to that seen in BAL of i.n. immunized mice.

### S.l. Administration of rADV induced SARS-CoV nAb

**Figure 3:**
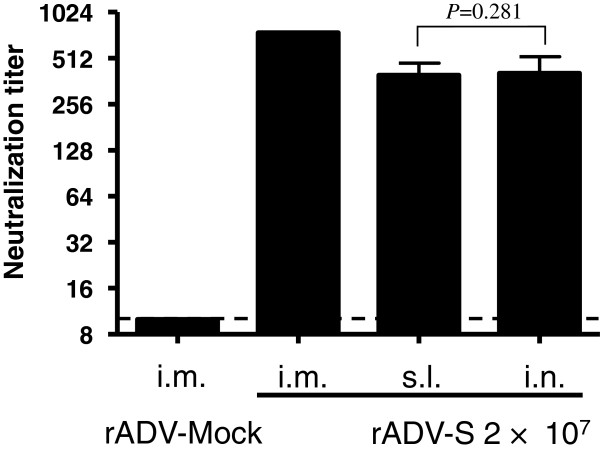
**SARS-CoV neutralizing activity of Sera.** Mice were immunized three times with rADV-S 2 × 10^7^ PFU by s.l., i.n., or i.m. route. Sera were tested to measure the Abs that would neutralize the infectivity of 100 TCID_50_ of SARS-CoV in Vero cell monolayers. Neutralization titers were determined by the CPE of SARS-CoV on Vero cell. The dotted line indicates the limitation of detection. The results are expressed as the means + SD.

nAbs against the S protein are considered a surrogate of protection against infection with the SARS-CoV [39,40]. We examined whether s.l. immunization with rADV-S induces nAbs. Groups of 6 mice each were immunized three times 2 weeks apart with rADV-S via either s.l., i.n., or i.m. route. Two weeks after the third immunization sera were collected and analyzed for SARS-CoV neutralization using microneutralization assay. As shown in Figure 3, all immunization routes induced significant levels of nAbs. The endpoint titers of nAbs are far above the titer of 1/35 that is considered to be protective in mice [36]. Thus, the results demonstrate that s.l. immunization with rADV-S induced high titer of nAbs against SARS-CoV infection, presumably protection against infection with SARS.

### S.l. Administration of rADV expressing SARS-CoV S induced virus-specific CD8^+^ T cell responses in the respiratory tract

**Figure 4:**
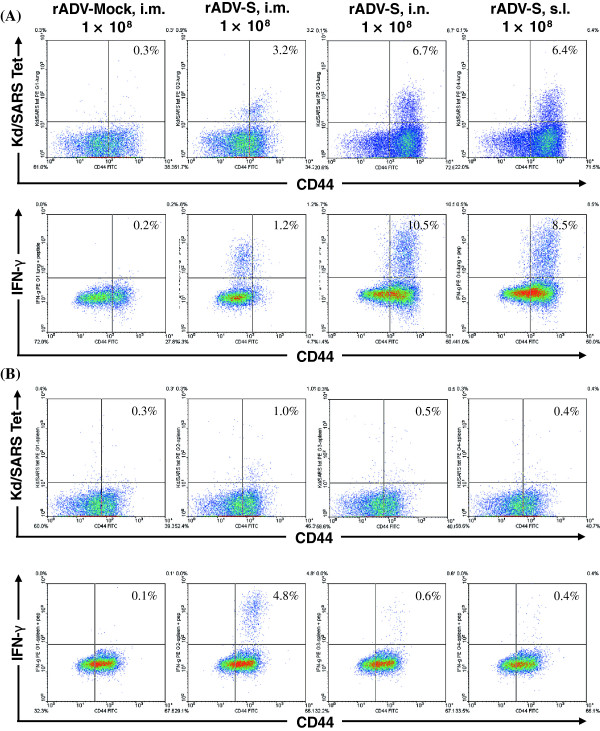
**Detection of S**_**366-374**_**epitope-specific and IFN-γ secreting CD8**^**+**^**T lymphocytes in the mice vaccinated with rADV-S.** Mice were immunized three times with rADV-S 1 × 10^8^ PFU by s.l., i.n., or i.m. route. Lung (**A**) and spleen (**B**) lymphocytes were harvested 10 days after the final immunization and were tested by flow cytometric analysis after staining with S_366-374_ tetramer, IFN-γ, CD8 and CD44.

It has been suggested that CD8^+^ T cell responses are important for clearance of other coronaviruses such as mouse hepatitis virus [41,42]. Therefore, we examined whether the administration of rADV-S via s.l., i.n., or i.m. route induces CD8^+^ T cell responses in the lungs and spleens. Ten days after the third vaccination, antigen-specific CD8^+^ T cells binding to MHC I tetramers containing the H-2K^d^-restricted SARS-CoV S epitope_366-374_ (CYGVSATKL) [43] and producing intracellular IFN-γ after *in vitro* re-stimulation with CYGVSATKL peptide were determined. As shown in Figure 4A, i.n. and s.l. immunization routes induced significantly higher percentages of SARS S-specific CD8^+^ T cells in the lungs (6.7 and 6.4%, respectively) as compared to i.m route (3.2%). Similarly, i.n. and s.l. immunization routes induced significantly higher percentages of IFN-γ-producing CD8^+^ T cells in the lung (10.5 and 8.5%, respectively) in response to SARS S protein. As expected, i.n. and s.l. immunization routes induced lower percentages of SARS S-specific CD8^+^ T cell and IFN-γ-producing CD8^+^ T cell in the spleens (Figure 4B) as compared to that induced by i.m. immunization. The results clearly demonstrate that s.l. and i.n. administrations of rADV-S are equally efficient in induction of CD8^+^ T cell responses in the lungs.

### S.l. Administration of rADV did not redirect virus to olfactory bulb

It has been reported that the i.n. administration of rADV resulted in virus migration to the olfactory bulb [7]. To investigate whether or not s.l. administration of rADV redirects the virus vector to the olfactory bulb, 1 × 10^8^ PFU of rADV were administered either intranasally or sublingually. Twenty-four hours later, the olfactory bulbs were collected and the presence of adenoviral DNA was determined by PCR. As shown in Table 1, no adenoviral DNA was detected in olfactory bulbs of s.l. immunized mice. In contrast, adenoviral DNA was detected in olfactory bulbs of all 8 i.n. immunized mice. Viral DNA was detected in the lungs of all s.l. immunized mice, as was the case for mice immunized intranasally.

**Table 1:** The distribution of rADV-EGFP in the lung and olfactory bulb of mice after i.n. or s.l. administration

**Route**	**PCR positive in lung**^**a**^	**PCR positive in olfactory bulb**
i.n.	8/8	8/8
s.l.	8/8	0/8

^a^ BALB/c mice were immunized with rADV-EGFP by i.n. or s.l. injection and next day, the lung and olfactory bulb were removed from the immunized mice. DNA was purified from the lungs and olfactory bulbs by using DNeasy tissue kit. The results were determined by PCR as described in Materials and Methods.

## Discussion

In this study, we demonstrate that s.l. administration of the rADV-S induced SARS-CoV S-specific immune responses in mucosal and systemic compartments. The results are in the line of our previous studies and those of others indicating that s.l. immunization induced broad spectrum of specific immune responses [30,31]. Indeed, s.l. immunization induced immune responses in respiratory [30] and vaginal tracts [44-46]. The former is not exceptional for our ADV encoding SARS-CoV since the rADV-S induced significant immune responses in respiratory tracts and systemic compartments. It is noteworthy that protein S specific IgA Ab response in BAL could be only induced when the animals were immunized either intranasally or sublingually. This characteristic of rADV is particularly important for the development of mucosal vaccines against respiratory pathogens. The levels of IgG and IgA induced in the blood upon s.l. immunization are comparable to those elicited by i.n. immunization which has been considered to be the best immunization route for induction of broad mucosal and systemic immune responses [30], indicating that s.l. immunization with rADV-S is an option for effective induction of systemic immunity. Importantly, s.l. immunization with rADV-S induced nAb at the level that is presumably protective against the challenge with live virus. It has been reported that nAbs targeting S protein play an important role in protection against SARS-CoV infection [47]. We found all immunization routes including s.l. immunization induced nAb titers above 1/35 that is considered to be protective in mice [36], indicating that our designed rADV-S is suitable for induction of protective immunity to wild type SARS-CoV.

In consideration of the emergence of nAb escape mutant and clinical observations in SARS patients who showed a decrease of CD8^+^ memory T cells [48], it is desirable to develop a SARS vaccine which can induce both humoral and cellular immune responses. Here, we showed that s.l. and i.n. immunizations of rADV-S induced higher levels of SARS-CoV S-specific CD8 T cell responses in the lung than that of i.m. immunization. The findings are in consistence with other studies showing that rADV expressing S protein could induce cellular immune responses [49,50]. Thus our immunization strategy involving rADV-S delivery through s.l. mucosa offers readily a tool to combat potential newly emerging SARS mutant.

There is a concern about pre-existing Abs against adenovirus in the human population that may prevent the immunization with rADV vector [51]. We showed that S-specific IgG Ab responses induced after the first immunization could be further boosted by a second administration of the rADV vector vaccine, but the third round of vaccination failed to induce an increase in the serum Ab levels. It is likely that the IgG titers induced after the second immunization already reached their maximum and could not be further boosted. Similarly, higher levels of serum specific IgG induced upon first i.m. immunization as compared to that induced by i.n. or s.l. immunization could not be further boosted.

It has been shown that i.n. but not i.m. immunization with rADV encoding SARS S and nucleocapsid (N) proteins significantly reduced SARS-CoV titer in the lungs after challenge, suggesting that i.n. immunization induced protective immune responses in the lungs, the site of infection [3]. Although i.n. delivery of vaccines induces effectively protective immunity, several observations raised safety concerns for its use in humans [7,25]. I.n. delivery of protein antigen together with CT as an adjuvant redirects antigen to CNS [31] and i.n. delivery of inactivated influenza vaccine caused Bell’s palsy in some human recipients [52]. In addition, i.n. delivery of rADV redirects the virus to the olfactory bulb via retrograde transport. These concerns call for an alternative immunization route for safe and effective induction of mucosal immune responses. Oral vaccination has been known to induce mucosal immunity and is safer than i.n. immunization, however degradation of the vaccine by the acidic pH and proteolytic enzymes in the gastrointestinal tract complicate the oral vaccination [2]. We chosen s.l. route as it was used for allergen immunotherapy [26] and was shown to be safe.

The s.l. delivery of rAd vectors expressing HIV-Gag was shown to induce Ag-specific CTL responses in mice even with preexisting immunity to Ad5 [33]. In addition, a recent study demonstrated that s.l. administration of rAd5 vector expressing HIV-Env was effective in penetrating the sublingual epithelium and induced Ag-specific mucosal Ab responses without adjuvant [32].

The present study describes a finding that s.l. delivery of rADV-S could induce systemic IgG and airway IgA SARS-CoV nAb as well as CD8^+^ T cell responses in mice. In contrary to i.n. administration, s.l. delivery of rADV does not redirect the rADV to the CNS. Our data indicated that s.l. administration could be an alternative mucosal route for safe and effective vaccination with rADV.

## Conclusions

The s.l. delivery of rADV-S induced humoral and cellular immune responses without accumulation of rADV in olfactory bulb. Importantly, these immune responses are comparable with those induced by i.n. administration. Thus, our study suggests that s.l. immunization with rADV-S offers a novel safe and effective vaccination strategy to combat SARS-CoV.

## Materials and methods

### Construction of rADV expressing SARS-CoV S protein

The ectodomain (amino acids 14–891) of the SARS-CoV S protein lacking the transmembrane domain and the cytoplasmatic tail was codon-optimized for high-level expression in mammalian cells and synthesized by GenScript Co. (Piscataway, NJ). The natural signal sequence was replaced by that of tissue plasminogen activator. Helical regions together with transmembrane domain and cytoplasmic domain were deleted as previously reported [39]. Briefly, rADV expressing SARS-CoV S gene was generated using the AdEasy Vector System according to the manufacturer’s instructions (Stratagene, La Jolla, CA). To inhibit the expression of SARS-CoV S gene during the course of ADV production, a tetracycline-regulated expression system was adopted. Two tetracycline operator sequences derived from pcDNA4/TO (Invitrogen, Carlsbad, CA) have been inserted between the TATA box of the CMV promoter of pShuttle-CMV (Stratagene), resulting in pShuttle-TetO2. After subcloning of SARS-CoV S gene into pShuttle-TetO2 (Figure 1A), this was co-transformed with adenoviral backbone vector, pAdEasy, into *Escherichia coli* (*E. coli*) BJ5183 by electroporation to achieve homologous recombination. The resulting construct was transfected into T-Rex-293 cells (Invitrogen) by the calcium phosphate co-precipitation method. T-Rex-293 cells were maintained according to the manual. Recombinant ADV was isolated from a single plaque, expanded in T-Rex-293 cells, and purified by double cesium chloride ultracentrifugation. The purified viruses were extensively dialyzed against 10 mM Tris, 5% sucrose, 2 mM MgCl_2_ and stored in aliquots at −80°C until use. Titers of ADV were determined by Tissue culture infectious dose 50 (TCID_50_) and by plaque assays in T-Rex-293 cells.

### Western blot analysis

The rADV-S infected 293 T cell lysate was separated by 10% SDS-PAGE. After electrophoretic transfer to nitrocellulose membrane (Schleicher & Schuell, Germany), the membrane was blocked with Tris-buffered saline (TBS) containing 5% skim milk and incubated with rabbit anti-SARS-S1 Ab (kindly provided by Chiron/Novartis, Italy) at a 1:3,000 dilution in TBST (TBS and 0.05% Tween 20) containing 5% skim milk for 1 hr at room temperature. After washing with TBST, the membranes were probed with incubation with goat-anti-rabbit Immunoglobulin G (IgG) conjugated to horseradish peroxidase (Santa Cruz Biotechnology, Santa Cruz, CA) at a 1:3,000 dilution in TBST containing 5% skim milk and detected by chromogenic substrate (ECL kit; Amersham Pharmacia Biotech Inc., Piscataway, NJ).

### Immunizations

The immunization schedule is summarized in Table 2. Six-week-old female BALB/c mice (Orient, Korea) were maintained under specific pathogen-free conditions and all studies were approved by Institutional Animal Care and Use Committee (IACUC) at the International Vaccine Institute (2010–015). Mice were immunized i.m. with rADV-S 2 × 10^7^ PFU in 100 μl phosphate-buffered saline (PBS), i.n. or s.l. with rADV-S 2 × 10^7^ or 1 × 10^8^ PFU in 20 μl of PBS [30]. In each experiment, mice (n = 6) were immunized three times at 2-week intervals. Control mice were immunized intramuscularly with rADV-Mock (2 × 10^7^ PFU/mouse).

**Table 2:** Immunization schedule

**Group**	**1st (day 0)**	**2nd (day 14)**	**3rd (day 28)**	**Routes**^**a**^
1	rADV-Mock	rADV-Mock	rADV-Mock	i.m.
2	rADV-S 2 × 10^7^	rADV-S 2 × 10^7^	rADV-S 2 × 10^7^	i.m.
3	rADV-S 2 × 10^7^	rADV-S 2 × 10^7^	rADV-S 2 × 10^7^	i.n.
4	rADV-S 1 × 10^8^	rADV-S 1 × 10^8^	rADV-S 1 × 10^8^	i.n.
5	rADV-S 2 × 10^7^	rADV-S 2 × 10^7^	rADV-S 2 × 10^7^	s.l.
6	rADV-S 1 × 10^8^	rADV-S 1 × 10^8^	rADV-S 1 × 10^8^	s.l.

^a^ Groups of six BALB/c mice were immunized s.l., i.n., or i.m. with rADV-S three times at 2-week intervals.

### Sample collection

Blood was collected from the retro-orbital plexus 2 weeks after each immunization, followed by incubation at room temperature for 30 min. Sera were obtained from the blood by centrifugation for 10 min at 13,000 rpm. Bronchoalveolar lavages (BAL) were collected on day 43 under anesthesia by repeated intra-tracheal flushing and aspiration of 500 μl of PBS per lung of mouse.

### ELISA

SARS-CoV S-specific Ab titers were determined by enzyme-linked immunosorbent assay (ELISA). Truncated SARS-CoV S protein (amino acids 201–510) was used as antigen. The gene encoding truncated S (residues 201–510) was inserted into pET15b vector (Novagen, Madison, WI). The protein was expressed in *E. coli* BL21 (DE3) (Novagen) and purified by Talon metal affinity column (Clontech, Palo Alto, CA). To measure the Ab responses, either the purified SARS-CoV S1 protein (amino acids 201–510) or a truncated S protein with a transmembrane deletion (Protein sciences corporation, Meriden, CT) was diluted to 2 μg/ml with 50 mM Sodiumbicarbonate buffer (pH 9.6). Microtiter plates (Nunc, Denmark) were pre-coated with 100 μl of the diluted protein per well and incubated overnight at 4°C. The plates were washed with PBS and blocked with 5% skim milk in PBS for 1 hr at room temperature. 100 μl of 2-fold serial dilution of samples in blocking buffer were added to each well and incubated for 1 hr at 37°C, followed by the addition of 1:3,000 diluted horseradish peroxidase-conjugated goat anti-mouse IgG or IgA (Santa Cruz biotechnology). After incubation for 1 hr at room temperature, 100 μl of peroxidase substrate tetramethylbenzidine (TMB) (Millipore, Bedford, MA) was added to each well. The reaction was stopped by adding 0.5 N HCl. The absorbance at wavelength 450 nm was recorded by a microplate reader (Molecular Devices, Sunnyvale, CA). The endpoint titer was determined by O.D. cut-off values of 0.2.

### Virus neutralization assay

The neutralization assay using active SARS-CoV was carried out in a biosafety level 3 laboratory. Virus microneutralization assay was performed as described previously [35]. Briefly, two-fold serial dilutions of heat-inactivated (30 min., 56°C) sera were tested against 100 TCID_50_ of SARS-CoV in Vero cell monolayers. The cytopathic effect (CPE) of SARS-CoV on Vero cell monolayers was read on day 4 and the neutralization titer was calculated by the Spearman/Karber formula [53].

### Flow cytometry analyses

For MHC class I tetramer staining, recombinant MHC class I Kd/SARS-CoV complexes were generated using the procedure described by D. Busch and E. Pamer (Yale University, New Haven, CT) [54]. Briefly, H-2K^d^ heavy chain-biotinylation site fusion and human β2-microglobulin were expressed in *E. coli*, purified from inclusion bodies, solubilized, and refolded in the presence of corresponding CYGVSATKL (S366-374) SARS-CoV peptide, a major CD8^+^ T cell epitope [43]. Complexes were then enzymatically biotinylated by BirA ligase (Avidity, Denver, CO) and were purified by Superdex-75 gel filtration and Mono-Q anion exchange chromatography (Amersham Pharmacia Biotech Inc.). The biotinylated monomer complexes were tetramerized with PE-labeled streptavidin (Molecular Probes, Eugene, OR). Tetramers were stored at 5 mg/ml in PBS (pH 8.0) containing 0.02% sodium azide, 1 μg/ml pepstatin, 1 μg/ml leupeptin, and 0.5 mM EDTA at 4°C. The lungs were perfused with 5 ml of PBS containing 10 U/ml heparin (Sigma-Aldrich) through the right ventricle using a syringe fitted with 25-gauge needle. The lungs were then removed and placed into RPMI medium supplemented with glutamine, gentamicin, penicillin G, and 10% fetal bovine serum (FBS) (HyClone, Logan, UT). The tissues were then processed through a steel screen to obtain single cell suspension, and particulate matter was removed by passing through 70 μm Falcon cell strainer (BD Labware). Freshly explanted lung cells were then purified by Percoll density gradient centrifugation. Spleen cells from immunized mice were resuspended in complete IMDM at a concentration of 1 × 10^7^ cells/ml. A total of 100 μl of these cells (1 × 10^6^ cells) were stained for CD8 (clone 53–6.7), CD44 (clone IM7), and Kd/SARS-CoV tetramer and samples were acquired on FACSCalibur (BD Biosciences, San Jose, CA). For intracellular staining, the cells were first stained for CD8 and CD44, washed, fixed and permeabilized with FACS buffer containing 0.5% saponin (Sigma-Aldrich). Then, the cells were stained with PE-conjugated anti-IFN-γ (XMG1.2) (eBioscience, Inc., San Diego, CA) or its control isotype Ab (rat IgG1) (eBioscience). Gates were set on lymphocytes by forward and side scatter profiles, and the data were analyzed using WinMDI version 2.9 software (The Scripps Research Institute, La Jolla, CA).

### Detection of DNA in tissues

Mice were administered either i.n. or s.l. with 1 × 10^8^ PFU of rADV-EGFP in 20 μl PBS. The olfactory bulbs and lungs were removed from the mice 24 hrs after the administration of rADV-EGFP. DNA was isolated using the DNeasy Tissue Kit (Qiagen, Valencia, CA) according to the manufacturer’s protocol.

The EGFP gene was amplified by polymerase chain reaction (PCR) using a forward primer (5’-CCGGGGATCCGGTGAGCAAGGGCGAGGAG-3’) and a reverse primer (5’-CCGGAAGCTTTCTTGTACAGCTCGTCCAT-3’). PCR was performed under the following conditions: 5 min at 95°C denaturation, 30 cycles: 30s at 95°C, 30s at 55°C, 1 min at 72°C; 10 min at 72°C additional extension. The PCR products were loaded on 1% agarose gel.

### Statistical analysis

Statistical differences between experimental and control groups were determined by unpaired Student’s *t* test. A *P* value of less than 0.05 was considered significant.

## Abbreviations

BAL: Bronchoalveolar lavages; CNS: Central nervous system; i.n: Intranasal; i.m: Intramuscular; PFU: Plaque-forming unit; rADV: Recombinant adenovirus; SARS-CoV: Severe acute respiratory syndrome-associated coronavirus; s.l.: Sublingual; TCID: Tissue culture infectious dose.

## Competing interests

The authors declare that they have no conflict of interest.

## Authors’ contributions

BSS carried out laboratory experiments and prepared the manuscript. KS carried out neutralization assay and participated in revising the manuscript. JC carried out FACS analysis and prepared the manuscript. HHN provided ideas and comments during manuscript preparation. CHY and DWK participated in revising the manuscript. CC and MKS conceived the experimental design and participated in revising the manuscript. All authors read and approved the final manuscript.

## References

[B1] Osek J Truszczynski M [Mucosal immunity with implications for use in developing a new generation of vaccines] Postepy Hig Med Dosw 1995 49 469 486 8657643

[B2] Holmgren J Czerkinsky C Mucosal immunity and vaccines Nat med 2005 11 S45 S53 10.1038/nm1213 15812489

[B3] See R Zakhartchouk A Petric M Lawrence D Mok C Hogan R Rowe T Zitzow L Karunakaran K Hitt M Comparative evaluation of two severe acute respiratory syndrome (SARS) vaccine candidates in mice challenged with SARS coronavirus J Gen Virol 2006 87 641 650 10.1099/vir.0.81579-0 16476986

[B4] Armstrong ME Lavelle EC Loscher CE Lynch MA Mills KH Proinflammatory responses in the murine brain after intranasal delivery of cholera toxin: implications for the use of AB toxins as adjuvants in intranasal vaccines J Infect Dis 2005 192 1628 1633 10.1086/491739 16206078

[B5] Fujihashi K Koga T van Ginkel FW Hagiwara Y McGhee JR A dilemma for mucosal vaccination: efficacy versus toxicity using enterotoxin-based adjuvants Vaccine 2002 20 2431 2438 10.1016/S0264-410X(02)00155-X 12057597

[B6] van Ginkel FW Jackson RJ Yuki Y McGhee JR Cutting edge: the mucosal adjuvant cholera toxin redirects vaccine proteins into olfactory tissues J Immunol 2000 165 4778 4782 11045998 10.4049/jimmunol.165.9.4778

[B7] Lemiale F Kong W Akyurek L Ling X Huang Y Chakrabarti B Eckhaus M Nabel G Enhanced mucosal immunoglobulin A response of intranasal adenoviral vector human immunodeficiency virus vaccine and localization in the central nervous system J Virol 2003 77 10078 10087 10.1128/JVI.77.18.10078-10087.2003 12941918 PMC224584

[B8] Tims T Briggs DJ Davis RD Moore SM Xiang Z Ertl HC Fu ZF Adult dogs receiving a rabies booster dose with a recombinant adenovirus expressing rabies virus glycoprotein develop high titers of neutralizing antibodies Vaccine 2000 18 2804 2807 10.1016/S0264-410X(00)00088-8 10812222

[B9] Sullivan NJ Sanchez A Rollin PE Yang ZY Nabel GJ Development of a preventive vaccine for Ebola virus infection in primates Nature 2000 408 605 609 10.1038/35046108 11117750

[B10] Xiang ZQ Yang Y Wilson JM Ertl HC A replication-defective human adenovirus recombinant serves as a highly efficacious vaccine carrier Virology 1996 219 220 227 10.1006/viro.1996.0239 8623532

[B11] Liu RY Wu LZ Huang BJ Huang JL Zhang YL Ke ML Wang JM Tan WP Zhang RH Chen HK Adenoviral expression of a truncated S1 subunit of SARS-CoV spike protein results in specific humoral immune responses against SARS-CoV in rats Virus Res 2005 112 24 31 10.1016/j.virusres.2005.02.009 16022898 PMC7114075

[B12] Tucker S Tingley D Scallan C Oral adenoviral-based vaccines: historical perspective and future opportunity Expert Rev Vaccines 2008 7 25 31 10.1586/14760584.7.1.25 18251691

[B13] Scott R Dudding B Romano S Russell P Enteric immunization with live adenovirus type 21 vaccine II. systemic and local immune responses following immunization Infect Immun 1972 5 300 304 4629075 10.1128/iai.5.3.300-304.1972 PMC422365

[B14] Yang T Millar J Grinshtein N Bassett J Finn J Bramson J T-cell immunity generated by recombinant adenovirus vaccines Expert Rev Vaccines 2007 6 347 356 10.1586/14760584.6.3.347 17542750

[B15] Hsu K Lubeck M Bhat B Bhat R Kostek B Selling B Mizutani S Davis A Hung P Efficacy of adenovirus-vectored respiratory syncytial virus vaccines in a new ferret model Vaccine 1994 12 607 612 10.1016/0264-410X(94)90264-X 8085377

[B16] Shanley J Wu C Intranasal immunization with a replication-deficient adenovirus vector expressing glycoprotein H of murine cytomegalovirus induces mucosal and systemic immunity Vaccine 2005 23 996 1003 10.1016/j.vaccine.2004.07.041 15620472

[B17] Shi Z Zeng M Yang G Siegel F Cain L Van Kampen K Elmets C Tang D Protection against tetanus by needle-free inoculation of adenovirus-vectored nasal and epicutaneous vaccines J Virol 2001 75 11474 11482 10.1128/JVI.75.23.11474-11482.2001 11689629 PMC114734

[B18] Morin J Lubeck M Barton J Conley A Davis A Hung P Recombinant adenovirus induces antibody response to hepatitis B virus surface antigen in hamsters Proc Natl Acad Sci USA 1987 84 4626 4630 10.1073/pnas.84.13.4626 2955413 PMC305143

[B19] Alkhatib G Briedis D High-level eucaryotic in vivo expression of biologically active measles virus hemagglutinin by using an adenovirus type 5 helper-free vector system J Virol 1988 62 2718 2727 3292790 10.1128/jvi.62.8.2718-2727.1988 PMC253705

[B20] Buge S Richardson E Alipanah S Markham P Cheng S Kalyan N Miller C Lubeck M Udem S Eldridge J An adenovirus-simian immunodeficiency virus env vaccine elicits humoral, cellular, and mucosal immune responses in rhesus macaques and decreases viral burden following vaginal challenge J Virol 1997 71 8531 8541 9343211 10.1128/jvi.71.11.8531-8541.1997 PMC192317

[B21] Dietzschold B Faber M Schnell MJ New approaches to the prevention and eradication of rabies Expert Rev Vaccines 2003 2 399 406 10.1586/14760584.2.3.399 12903805

[B22] Patel A Zhang Y Croyle M Tran K Gray M Strong J Feldmann H Wilson JM Kobinger GP Mucosal delivery of adenovirus-based vaccine protects against Ebola virus infection in mice J Infect Dis 2007 196 Suppl 2 S413 S420 17940978 10.1086/520603

[B23] Xiang Z Li Y Gao G Wilson JM Ertl HC Mucosally delivered E1-deleted adenoviral vaccine carriers induce transgene product-specific antibody responses in neonatal mice J Immunol 2003 171 4287 4293 14530353 10.4049/jimmunol.171.8.4287

[B24] Damjanovic D Zhang X Mu J Fe Medina M Xing Z Organ distribution of transgene expression following intranasal mucosal delivery of recombinant replication-defective adenovirus gene transfer vector Genet Vaccines Ther 2008 6 5 10.1186/1479-0556-6-5 18261231 PMC2259349

[B25] Davidson B Allen E Kozarsky K Wilson J Roessler B A model system for in vivo gene transfer into the central nervous system using an adenoviral vector Nat Genet 1993 3 219 223 10.1038/ng0393-219 8387378

[B26] Burastero S Mistrello G Falagiani P Paolucci C Breda D Roncarolo D Zanotta S Monasterolo G Rossi R Effect of sublingual immunotherapy with grass monomeric allergoid on allergen-specific T-cell proliferation and interleukin 10 production Ann Allergy Asthma Immunol 2008 100 343 350 10.1016/S1081-1206(10)60597-2 18450120

[B27] Akdis M Immunologic responses to sublingual allergen immunotherapy Clin Allergy Immunol 2008 21 71 86 18828499

[B28] Bohle B Kinaciyan T Gerstmayr M Radakovics A Jahn-Schmid B Ebner C Sublingual immunotherapy induces IL-10-producing T regulatory cells, allergen-specific T-cell tolerance, and immune deviation J Allergy Clin Immunol 2007 120 707 713 10.1016/j.jaci.2007.06.013 17681368

[B29] Madonini E Agostinis F Barra R Berra A Donadio D Pappacoda A Stefani E Tierno E Long-term and preventive effects of sublingual allergen-specific immunotherapy: a retrospective, multicentric study Int J Immunopathol Pharmacol 2003 16 73 79 12578735 10.1177/039463200301600111

[B30] Cuburu N Kweon M Song J Hervouet C Luci C Sun J Hofman P Holmgren J Anju re F Czerkinsky C Sublingual immunization induces broad-based systemic and mucosal immune responses in mice Vaccine 2007 25 8598 8610 10.1016/j.vaccine.2007.09.073 17996991

[B31] Song J Nguyen H Cuburu N Horimoto T Ko S Park S Czerkinsky C Kweon M Sublingual vaccination with influenza virus protects mice against lethal viral infection Proc Natl Acad Sci USA 2008 105 1644 1649 10.1073/pnas.0708684105 18227512 PMC2234198

[B32] Domm W Brooks L Chung HL Feng C Bowers WJ Watson G McGrath JL Dewhurst S Robust antigen-specific humoral immune responses to sublingually delivered adenoviral vectors encoding HIV-1 Env: Association with mucoadhesion and efficient penetration of the sublingual barrier Vaccine 2011 29 7080 7089 10.1016/j.vaccine.2011.07.008 21801777 PMC3167942

[B33] Appledorn DM Aldhamen YA Godbehere S Seregin SS Amalfitano A Sublingual administration of an adenovirus serotype 5 (Ad5)-based vaccine confirms toll-like receptor agonist activity in the oral cavity and elicits improved mucosal and systemic cell-mediated responses against HIV antigens despite preexisting ad5 immunity Clin Vaccine Immunol 2011 18 150 160 10.1128/CVI.00341-10 21084461 PMC3019785

[B34] Traggiai E Becker S Subbarao K Kolesnikova L Uematsu Y Gismondo MR Murphy BR Rappuoli R Lanzavecchia A An efficient method to make human monoclonal antibodies from memory B cells: potent neutralization of SARS coronavirus Nat med 2004 10 871 875 10.1038/nm1080 15247913 PMC7095806

[B35] Subbarao K McAuliffe J Vogel L Fahle G Fischer S Tatti K Packard M Shieh W Zaki S Murphy B Prior infection and passive transfer of neutralizing antibody prevent replication of severe acute respiratory syndrome coronavirus in the respiratory tract of mice J Virol 2004 78 3572 3577 10.1128/JVI.78.7.3572-3577.2004 15016880 PMC371090

[B36] Bisht H Roberts A Vogel L Bukreyev A Collins P Murphy B Subbarao K Moss B Severe acute respiratory syndrome coronavirus spike protein expressed by attenuated vaccinia virus protectively immunizes mice Proc Natl Acad Sci USA 2004 101 6641 6646 10.1073/pnas.0401939101 15096611 PMC404098

[B37] Mitragotri S Immunization without needles Nat Rev Immunol 2005 5 905 916 10.1038/nri1728 16239901

[B38] Neutra M Kozlowski P Mucosal vaccines: the promise and the challenge Nat Rev Immunol 2006 6 148 158 10.1038/nri1777 16491139

[B39] Yang Z Kong W Huang Y Roberts A Murphy B Subbarao K Nabel G A DNA vaccine induces SARS coronavirus neutralization and protective immunity in mice Nature 2004 428 561 564 10.1038/nature02463 15024391 PMC7095382

[B40] Lee J Poo H Han D Hong S Kim K Cho M Kim E Sung M Kim C Mucosal immunization with surface-displayed severe acute respiratory syndrome coronavirus spike protein on Lactobacillus casei induces neutralizing antibodies in mice J Virol 2006 80 4079 4087 10.1128/JVI.80.8.4079-4087.2006 16571824 PMC1440448

[B41] Williamson J Stohlman S Effective clearance of mouse hepatitis virus from the central nervous system requires both CD4+ and CD8+ T cells J Virol 1990 64 4589 4592 2166833 10.1128/jvi.64.9.4589-4592.1990 PMC247935

[B42] Harty J Tvinnereim A White D CD8+ T cell effector mechanisms in resistance to infection Annu Rev Immunol 2000 18 275 308 10.1146/annurev.immunol.18.1.275 10837060

[B43] Zhi Y Kobinger G Jordan H Suchma K Weiss S Shen H Schumer G Gao G Boyer J Crystal R Identification of murine CD8 T cell epitopes in codon-optimized SARS-associated coronavirus spike protein Virology 2005 335 34 45 10.1016/j.virol.2005.01.050 15823604 PMC7111773

[B44] Cho H Kim J Lee Y Kim J Kim Y Chun T Oh Y Enhanced humoral and cellular immune responses after sublingual immunization against human papillomavirus 16 L1 protein with adjuvants Vaccine 2010 28 2598 2606 10.1016/j.vaccine.2010.01.013 20116467

[B45] Cuburu N Kweon MN Hervouet C Cha HR Pang YYS Holmgren J Stadler K Schiller JT Anjuere F Czerkinsky C Sublingual immunization with nonreplicating antigens induces antibody-forming cells and cytotoxic T cells in the female genital tract mucosa and protects against genital papillomavirus infection J Immunol 2009 183 7851 7859 10.4049/jimmunol.0803740 19933861 PMC7370923

[B46] Hervouet C Luci C Cuburu N Cremel M Bekri S Vimeux L Maranon C Czerkinsky C Sublingual immunization with an HIV subunit vaccine induces antibodies and cytotoxic T cells in the mouse female genital tract Vaccine 2010 28 5582 5590 10.1016/j.vaccine.2010.06.033 20600505

[B47] Du L He Y Zhou Y Liu S Zheng BJ Jiang S The spike protein of SARS-CoV–a target for vaccine and therapeutic development Nat Rev Microbiol 2009 7 226 236 10.1038/nrmicro2090 19198616 PMC2750777

[B48] Huang J Huang J Duan Z Wei J Min J Luo X Li J Tan W Wu L Liu R Th2 predominance and CD8+ memory T cell depletion in patients with severe acute respiratory syndrome Microbes Infect 2005 7 427 436 10.1016/j.micinf.2004.11.017 15784184 PMC7110803

[B49] See R Petric M Lawrence D Mok C Rowe T Zitzow L Karunakaran K Voss T Brunham R Gauldie J Severe acute respiratory syndrome vaccine efficacy in ferrets: whole killed virus and adenovirus-vectored vaccines J Gen Virol 2008 89 2136 2146 10.1099/vir.0.2008/001891-0 18753223

[B50] Kobinger G Figueredo J Rowe T Zhi Y Gao G Sanmiguel J Bell P Wivel N Zitzow L Flieder D Adenovirus-based vaccine prevents pneumonia in ferrets challenged with the SARS coronavirus and stimulates robust immune responses in macaques Vaccine 2007 25 5220 5231 10.1016/j.vaccine.2007.04.065 17559989 PMC7115643

[B51] Barouch DH McKay PF Sumida SM Santra S Jackson SS Gorgone DA Lifton MA Chakrabarti BK Xu L Nabel GJ Plasmid chemokines and colony-stimulating factors enhance the immunogenicity of DNA priming-viral vector boosting human immunodeficiency virus type 1 vaccines J Virol 2003 77 8729 8735 10.1128/JVI.77.16.8729-8735.2003 12885892 PMC167238

[B52] Lewis D Huo Z Barnett S Kromann I Giemza R Galiza E Woodrow M Thierry-Carstensen B Andersen P Novicki D Transient facial nerve paralysis (Bell's palsy) following intranasal delivery of a genetically detoxified mutant of Escherichia coli heat labile toxin PLoS One 2009 4 e6999 10.1371/journal.pone.0006999 19756141 PMC2737308

[B53] Hamilton MA Russo RC Thurston RV Trimmed Spearman-Karber method for estimating median lethal concentrations in toxicity bioassays Environ Sci Technol 1977 11 714 719 10.1021/es60130a004

[B54] Busch DH Pilip IM Vijh S Pamer EG Coordinate regulation of complex T cell populations responding to bacterial infection Immunity 1998 8 353 362 10.1016/S1074-7613(00)80540-3 9529152

